# Bone marrow-derived and resident liver macrophages display unique transcriptomic signatures but similar biological functions

**DOI:** 10.1016/j.jhep.2016.05.037

**Published:** 2016-10

**Authors:** Lynette Beattie, Amy Sawtell, Jason Mann, Teija C.M. Frame, Bianca Teal, Fabian de Labastida Rivera, Najmeeyah Brown, Katherine Walwyn-Brown, John W.J. Moore, Sandy MacDonald, Eng-Kiat Lim, Jane E. Dalton, Christian R. Engwerda, Kelli P. MacDonald, Paul M. Kaye

**Affiliations:** 1 Centre for Immunology and Infection, Hull York Medical School and Dept. of Biology, University of York, York YO10 5DD, UK; 2 Biosciences Technology Facility, Dept. of Biology, University of York, York YO10 5DD, UK; 3 QIMR Berghofer Medical Research Institute, 300 Herston Rd, Herston, Queensland 4006, Australia

**Keywords:** Kupffer cells, Liver macrophages, *Listeria*, *Neisseria*, *Salmonella*, *Leishmania*, Scavenger receptors

## Abstract

**Background & Aims:**

Kupffer cells (KCs), the resident tissue macrophages of the liver, play a crucial role in the clearance of pathogens and other particulate materials that reach the systemic circulation. Recent studies have identified KCs as a yolk sac-derived resident macrophage population that is replenished independently of monocytes in the steady state. Although it is now established that following local tissue injury, bone marrow derived monocytes may infiltrate the tissue and differentiate into macrophages, the extent to which newly differentiated macrophages functionally resemble the KCs they have replaced has not been extensively studied.

**Methods:**

We studied the two populations of KCs using intravital microscopy, morphometric analysis and gene expression profiling. An ion homeostasis gene signature, including genes associated with scavenger receptor function and extracellular matrix deposition, allowed discrimination between these two KC sub-types.

**Results:**

Bone marrow derived “KCs” accumulating as a result of genotoxic injury, resemble but are not identical to their yolk sac counterparts. Reflecting the differential expression of scavenger receptors, yolk sac-derived KCs were more effective at accumulating acetylated low density lipoprotein, whereas surprisingly, they were poorer than bone marrow-derived KCs when assessed for uptake of a range of bacterial pathogens. The two KC populations were almost indistinguishable in regard to i) response to lipopolysaccharide challenge, ii) phagocytosis of effete red blood cells and iii) their ability to contain infection and direct granuloma formation against *Leishmania donovani*, a KC-tropic intracellular parasite.

**Conclusions:**

Bone marrow-derived KCs differentiate locally to resemble yolk sac-derived KC in most but not all respects, with implications for models of infectious diseases, liver injury and bone marrow transplantation. In addition, the gene signature we describe adds to the tools available for distinguishing KC subpopulations based on their ontology.

**Lay summary:**

Liver macrophages play a major role in the control of infections in the liver and in the pathology associated with chronic liver diseases. It was recently shown that liver macrophages can have two different origins, however, the extent to which these populations are functionally distinct remains to be fully addressed. Our study demonstrates that whilst liver macrophages share many features in common, regardless of their origin, some subtle differences in function exist.

**Data repository:**

Gene expression data are available from the European Bioinformatics Institute ArrayExpress data repository (accession number E-MTAB-4954).

## Graphical abstract

**Figure:**
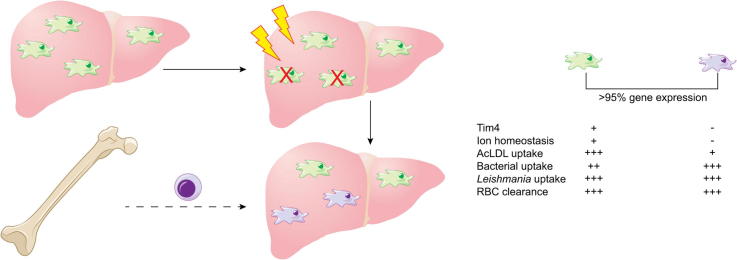

## Introduction

Kupffer cells (KCs), the resident tissue macrophages of the liver have a crucial role in both the pathogenesis and the resolution of various liver diseases and inflammatory states including alcohol-induced liver injury [1], non-alcoholic fatty liver disease associated with obesity [2], ischemia reperfusion injury [3], immune tolerance to organ transplantation [3] and infectious disease [4].

Resident tissue macrophages, including KCs, were historically considered a hematopoietic population, with replenishment of the tissue reservoir from monocyte-derived precursors in the steady state. This view has now been challenged with the majority of tissue macrophages shown to develop independently of haematopoietic stem cells, being seeded in the tissues prior to birth from a population of yolk sac (YS) derived macrophages [5], [6]. These cells show some level of radiation resistance [7] and are independent of replenishment by monocytes in the steady state [8], [9]. Parallel studies that identified the transcription factors MafB and c-Maf as the factors that control the self-renewal of differentiated macrophages [10], and the observation that tissue macrophages are capable of self-renewal in models of acute inflammation [11] and under T-helper cell 2 conditions in the presence of interleukin (IL)-4 [12], have confirmed that mature tissue macrophages are capable of proliferation and self-renewal. Finally, recent landmark studies have demonstrated that macrophage identity is unique to each macrophage population and is plastic, with phenotype conferred by microenvironment rather than cellular origin [13], [14], [15]. Together, the above studies represent a paradigm shift in the field of tissue macrophage biology.

The studies described above all examined the origin of tissue macrophages only in the steady state. However, there is clear evidence that infection or tissue injury, and the associated inflammatory response, promotes the recruitment of myeloid cells, mostly monocytes into peripheral tissues. It is unclear when, and if, the newly infiltrated monocytes undergo differentiation into macrophages *in situ*. In fact, some authors have described the infiltrating cells as tissue macrophages [16], sometimes as early as 24 h post infiltration [17] without defining any phenotypic or functional changes in the cells. Although once infiltrated, monocytes begin differentiating into cells that are similar to the macrophages in the tissue that they reside in [18], the function of these bone marrow (BM)-derived monocytes once they are present in the tissue has not been fully investigated. Given that KCs are implicated in the pathogenesis and the resolution of a number of liver diseases [2], [3], [4], [19], [20], [21] and their phagocytic capacity makes them an easy target for particle based therapeutics [22], a greater understanding of KC biology and heterogeneity will facilitate the development of targeted liver therapeutics. Understanding whether distinct functions can be attributed to KCs of different origin will also be important for the design of new anti-infective strategies.

Here, we have used an irradiation bone marrow chimera model to enforce loss of YS-derived KCs and repopulation of the KC niche from BM-derived precursors. Using intravital microscopy to characterise the morphological and dynamic properties of YS- and BM-derived KCs *in situ*, and microarray analysis to examine gene expression profile, we show that after 6 weeks of differentiation in the liver, BM-derived “KCs” closely resemble but are not fully identical to the YS-derived KCs they have replaced. Whilst uptake of acetylated low density lipoprotein (Ac-LDL) was more prominent in YS-derived macrophages and the converse was true for bacterial uptake, for most of the functional studies we performed, these populations were functionally similar. This was particularly notable in their capacity to exert early control of and direct granuloma formation in response to infection with the KC-tropic intracellular protozoan parasite *Leishmania donovani*. These findings demonstrate that in the context of enforced liver inflammation, BM derived monocytes transition into KCs, which are as capable of protecting the host from infectious challenge as their YS-derived counterparts.

## Materials and methods

### Mice and infection

C57BL6 or B6.CD45.1 mice were obtained from Charles River (UK) or the Australian Resource Centre (WA). mT/mG [1], lysMcre [2] and B6.MacGreen [3] mice have been previously described. Mice were bred and housed under specific pathogen-free conditions and used at 6–12 weeks of age. The Ethiopian strain of *Leishmania donovani* (LV9) and tandem Tomato fluorescent protein expressing LV9 (tdTom.LV9) [4] were maintained by serial passage in *Rag1*^−/−^ mice. Amastigotes were isolated from infected spleens [5], and mice were infected with 3 × 10^7^ *L. donovani* amastigotes intravenously (i.v.) via the tail vein in 200 μl of RPMI 1640 (GIBCO, UK). For the generation of chimeras, mice were placed on acidified water for at least 2 days prior to irradiation. Donor mice were irradiated with 1100 rads on a split-dose regimen (550 rads per dose, 24 h apart) and were then reconstituted with 2–5 × 10^6^ donor bone marrow cells via tail vein injection. Reconstituted mice were treated with oral antibiotics (Baytril) for 4 weeks post-reconstitution.

### Liver enzyme analysis

Heparinized blood was immediately centrifuged for 10 min at 300 g. Plasma was stored at −80 °C until analysis. Alanine aminotransferase (ALT) and aspartate transaminase (AST) levels were determined using a Beckman Unicell DxC800 analyzer in a single batch.

### Confocal microscopy

Confocal microscopy was performed on 20 μm frozen sections. For tissue containing tdTom expressing parasites, tissues were fixed in 4% paraformaldehyde (PFA) for two h before overnight incubation in 30% sucrose and embedding in OCT medium (Sakura). Antibodies were conjugated to Alexa488 or Alexa647 (eBioscience, UK). Slides were blinded before imaging on a Zeiss LSM510 axioplan microscope (Carl Zeiss Microimaging). Data were rendered and analysed using Volocity software (Improvision).

### Ethics statement

All experiments were approved by the University of York Animal Welfare and Ethical Review Body and performed under UK Home Office license (‘Immunity and Immunopathology of Leishmaniasis’ Ref # PPL 60/3708) or approved by the Queensland Institute of Medical Research Berghofer (QIMRB) animal ethics committee Ref #P2076 (A1412-614).

### Intravital imaging

Mice were anaethetised and surgery performed similar to previously described [23] except that anaethesia was maintained by inhalation of 4% isofluorane (Abbott laboratories, UK). Images were acquired on an inverted LSM 780 multiphoton microscope (Carl Zeiss Microimaging), maintained at 36 °C by a blacked-out environmental chamber (Solent Scientific, UK). Images were acquired with a 40x 1.1 water immersion objective and fluorescence excitation provided by a Chameleon XR Ti:sapphire laser (Coherent) tuned to 870 nm.

### Whole genome array

RNA was isolated from purified KC and amplified via Agilent low-input Quick Amp labelling kit (Agilent Technologies). Amplified RNA was then assayed with Agilent SurePrint G3 mouse GE 8 × 60 k microarray chips that were scanned with an Agilent C Scanner with Surescan High Resolution Technology (Agilent Technologies). The data were normalized using the percentile shift method to the 75^th^ percentile. Comparison of the gene expression data between liver resident and BM-derived KCs was performed using the Benjamini and Hochberg false discovery rate (FDR) correction [24]. This analysis was performed with GeneSpring software (version 9; Agilent) as a standard 5% FDR, with the variances assessed by the software for each *t* test performed. A 2-fold expression criterion was then applied to each gene list. Gene ontology analysis was performed using the GeneSpring (Agilent) and Ingenuity pathway systems analysis software packages (Ingenuity Systems). Gene expression data is available from European Bioinformatics Institute ArrayExpress (accession number E-MTAB-4954).

Further methodology may be found in the Supplementary materials and methods.

## Results

### Radiation-induced liver injury causes loss of a proportion of liver resident KCs and their replenishment from the bone marrow

**Fig. 1:**
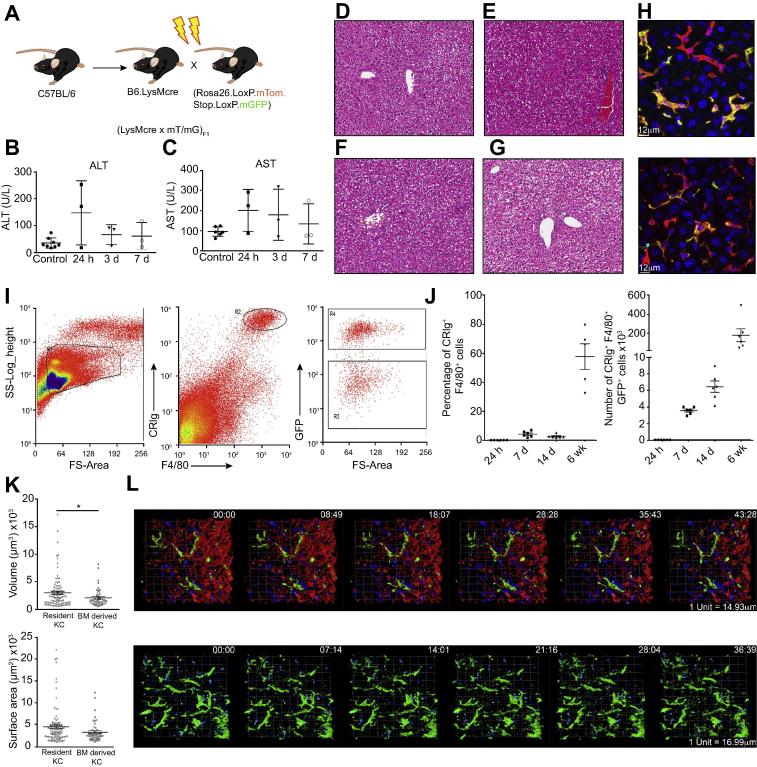
**Characterisation of YS- and BM-derived KCs.** (A) Experimental approach used to generate irradiation chimeras with GFP^+^ YS-derived KCs. (B) ALT and (C) AST levels in the serum of mice at different time points post-irradiation as compared to control (non-irradiated) animals. (D) H&E stained sections from the liver of control non-irradiated mice or mice (E) 24 h (F) 3 days or (G) 7 days post-irradiation demonstrating very little liver damage or inflammation as a result of irradiation. (H) Immunofluorescent image demonstrating the presence of GFP^+^ F4/80^+^ liver resident KCs and GFP^-^ F4/80^+^ BM-derived KC in the livers of the chimeras generated in (A) (left) or reciprocal chimeras (right), 6 weeks post-irradiation. GFP (green), F4/80 (red). (I) Flow cytometry analysis of the livers of chimeras generated via the method shown in (A) gated on forward scatter (FSC) and side scatter (SSC), CRIg and F4/80 expression and GFP. (J) Analysis of the percentage of CRIg^+^ F4/80^+^ cells that express GFP (left) and the number of CRIg^+^ F4/80^+^ GFP^+^ cells in the liver (right) over time. Symbols represent individual mice and are representative of 2 experiments with 5-6 mice per group. (I) The volume and surface area of KCs in 3 dimensions. (L) 2-photon intravital imaging of YS-derived KCs in the livers of chimeras generated via the method shown in (A) (top) or the reciprocal chimera (bottom). Data were analysed with a non-parametric *t* test. ∗*p* <0.05.

To study KCs, we used (LysM-Cre x mT/mG)_F1_ mice. To confirm that the majority of KCs expressed Cre recombinase and were therefore green fluourescent protein (GFP) positive in this system, we performed immunofluorescent staining on fixed liver tissue. By this assay, 95% ± 2.04% of F4/80^+^ KCs expressed GFP. Therefore, we used these mice in subsequent experiments. We generated BM chimeras by irradiating (LysM-Cre x mT/mG)_F1_ mice and reconstituting them with wild-type C57BL/6 BM (Fig. 1A). This process resulted in transient liver damage and inflammation as assessed by a small but non-significant increase in ALT and AST levels in the serum of irradiated mice compared to control, non-irradiated mice (Fig. 1B, C). Histological examination of H&E sections revealed detectable portal and lobular inflammation at 24 h (Fig. 1E) and 3 days post-irradiation in one out of 3 mice in each group (Fig. 1F), but this was no longer visible after 7 days (Fig. 1G).

A proportion of the YS-derived macrophages that express GFP were lost as a result of irradiation, and these cells were replaced by F4/80^+^ GFP^-^ BM-derived KCs in the chimeric mice at 6 weeks post-irradiation (Fig. 1H). Conversely, reciprocal (LysM-Cre x mT/mG)_F1_ →C57BL/6 chimeras resulted in a loss of a proportion of GFP^-^ YS-derived KCs and their replacement with BM-derived GFP^+^ F4/80^+^ KCs at 6 weeks post-irradiation (Fig. 1H). Flow cytometric analysis of digested livers (Fig. 1I) with gating on complement receptor of the immunoglobulin superfamily (CRIg)^+^ F4/80^hi^ cells (Fig. 1I) demonstrated that both GFP^+^ and GFP^-^ KCs were present in chimeric mice. Importantly, the generation of mixed BM chimeras in this way allows for analysis of the function of each population *in situ* under the same microenvironmental conditions.

We then studied the kinetics of KC depletion and repopulation after irradiation in (LysM-Cre x mT/mG)_F1_ → C57BL/6 chimeras. The percentage of GFP^+^ cells that express CRIg and F4/80 and the total number of CRIg^+^ F4/80^+^ GFP^+^ cells (Fig. 1J) increased over time after irradiation to reach approximately 50% of the KC population by 6 weeks post-irradiation. We next used 2-photon real-time *in vivo* imaging of these reciprocal chimeras (Fig. 1K and L) to examine the morphology and motility of each KC population *in situ*. In C57BL/6 → (LysM-Cre x mT/mG)_F1_ chimeras, GFP^+^ YS-derived KCs were large, interdigitating cells that were active in their membrane movements, but did not travel along the sinusoids (Fig. 1L; Supplementary movie 1). BM-derived KCs observed in (LysM-Cre x mT/mG)_F1_ → C57BL/6 chimeras also showed similar morphology and remained stationary within the sinusoids, confirming that they had become resident within the liver (Fig. 1L; Supplementary movie 2). Analysis of KC volume and surface area demonstrated that YS-derived KCs were somewhat larger than BM-derived KCs (Fig. 1K). Taken together, these data demonstrate that following genotoxic damage BM-derived cells differentiate morphologically into KCs and become resident in the liver.

### An ion homeostasis gene signature distinguishes tissue-resident from BM-derived KCs

**Fig. 2:**
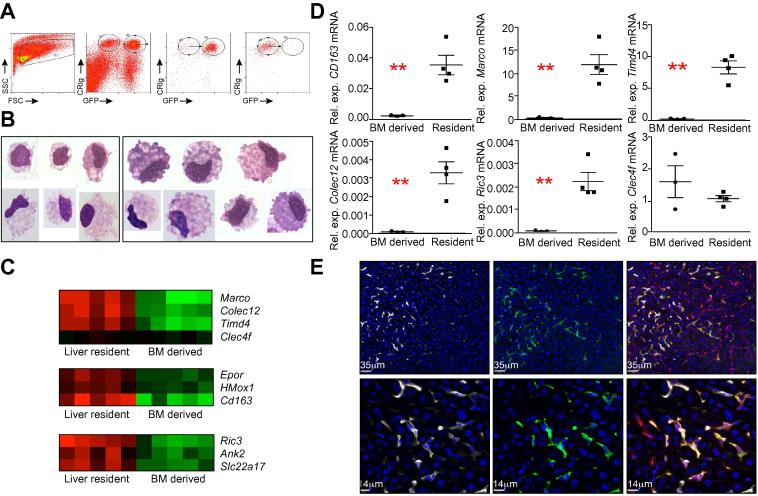
**Transcriptional analysis of YS- and BM-derived KCs.** (A) Isolation of YS-derived and BM-derived KCs by high speed fluorescence activated cell sorting according to forward and side scatter, GFP and CRIg. Post-sort purity of GFP^+^ and GFP^-^ KCs. (B) Giemsa stained cytospins of sorted BM-derived (left) or liver resident (right) KCs. (C) Heat maps demonstrating the differential binding to probe sets across the biological replicates for selected groups of genes. (D) Accumulation of mRNA for selected genes expressed as relative expression to hypoxanthine-guanine phosphoribosyltransferase (*HPRT*). Individual symbols are representative of KCs sorted from individual mice. Data were analysed using a non-parametric t test. ∗∗*p* <0.01. (E) Immunofluorescent images demonstrating the expression of MARCO (white) on GFP^+^ (green) F4/80^+^ (red) liver resident KCs at 200x (top) and 630x (bottom) magnification.

We next sought to determine whether BM-derived KCs had indeed acquired global characteristics of YS-derived KCs via a comparative gene expression approach. BM-derived and YS-derived KCs were isolated from chimeric mice by fluorescence activated high speed cell sorting according to size, granularity and expression of CRIg (Fig. 2A). KCs were then further separated into GFP^+^ and GFP^-^ cell fractions. Sorted populations were typically >90% pure for the population of interest. Giemsa stained cytopsin preparations of sorted cells demonstrated that BM-derived and YS-derived KCs had similar macrophage-like morphology (Fig. 2B). Gene expression analysis by microarray demonstrated that of the annotated genes that were represented on the chips, BM-derived and YS-derived KCs shared expression of >99% of the probes, with only 42 probes meeting criteria for differentially binding of the cDNA between the two populations (5% FDR with 2-fold cut-off, Table 1, Fig. 2C). Of note, the binding of these 42 transcripts was present for the resident KC population and lacking in BM-derived KCs. There were no transcripts uniquely present in BM- derived KCs. KCs showed similar transcript profiles whether isolated from chimeras that resulted in them being GFP^+^ or GFP^-^, with no significant differences in expression observed within the GFP^+^ and GFP^-^ fractions within the same KC population (data not shown).

Validation of differential expression of a proportion of the genes listed in Table 1 was then performed by measuring mRNA abundance by real-time PCR, using RNA isolated from independently sorted samples of each KC population. mRNA abundance for *Cd163*, *Marco*, *Ric3*, *Colec12* and *Timd4* (Fig. 2D) was higher in YS-derived KCs than in BM-derived KCs, confirming the microarray data (Fig. 2C). Abundance of *Clec4f* mRNA was similar between the populations, also confirming the microarray data and validating that a transition to KC, as defined by *Clec4f* (Kupffer Cell Receptor), had occurred in the BM-derived KC population.

Macrophage Receptor with Collagenous Structure (MARCO) was one of the most differentially expressed genes found in YS-derived KCs, with 15-fold higher mRNA abundance than seen in BM-derived KCs. We therefore analysed the expression of MARCO protein in KCs in C57BL/6 → (LysM-Cre x mT/mG)_F1_ chimeras. Clear co-expression of MARCO and GFP was observed in YS-derived KCs, but very little detectable expression of MARCO in GFP^-^ BM-derived KCs (Fig. 2E). In further validation experiments, flow cytometry on isolated hepatic mononuclear cells from B6.MacGreen → C57BL/6 chimeras demonstrated that Tim4 was expressed in 73.4 ± 4.21% of YS-derived KCs and only expressed in 5 ± 0.26% of BM-derived KCs confirming that Tim4 expression is enriched within the YS-derived KC population.

To gain a better understanding of the implications of the transcriptomic differences between YS-derived and BM-derived KCs, we performed gene ontology (GO) analysis. 3 of the 4 GO terms that showed highly significant enrichment (*p* <0.001) were associated with ion homeostasis (Supplementary Table 1). This included GO:0055065; metal ion homeostasis, GO:0055080; cation homeostasis and GO:0050801; ion homeostasis. This enrichment was associated specifically with the expression of *Ric3*, *Ank2*, *Slc22a17*, *Epor* and *Hmox1* in YS-derived KCs (Fig. 2C). Resistant to inhibitors of cholinesterase 3 (Ric3) is a transmembrane protein that controls expression of nicotinic acetylcholine receptors, which are gated ion channels [25]. Ankyrin 2 (Ank2) is also associated with ion channels, being an adaptor protein for connection of ion channels to the actin cytoskeleton [26]. Slc22a17 (also known as 24p3R) is associated with iron uptake and apoptosis [27]. In addition, mRNA for the erythropoietin receptor (*Epor*), hemeoxygenase 1 (*Hmox1*), both associated with red blood cell homeostasis and the haemoglobin scavenger receptor *Cd163* [28] were more abundant in YS-derived KCs, the latter being one of the most differentially expressed genes (Fig. 2C).

### BM-derived and YS-derived KCs exhibit comparable clearance of effete red blood cells

**Fig. 3:**
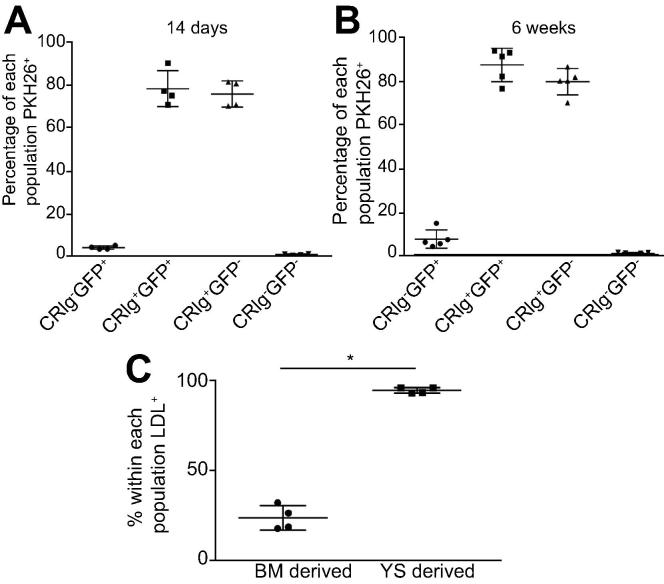
**YS and BM-derived KCs have similar capacity to clear neuraminidase treated labelled red blood cells.** C57BL/6 recipient mice received B6.mTmG.LysM^Cre^ bone marrow and PKH26 labelled red blood cells (A) two weeks or (B) 6 weeks post-irradiation. Hepatic mononuclear cells were prepared and plots gated on CRIg^+^ and GFP expression to examine four populations. CRIg^-^GFP^+^ BM-derived cells. CRIg^+^GFP^+^ differentiated BM- derived KCs. CRIg^+^GFP^−^ YS-derived KCs and CRIg^-^GFP^-^ non-macrophage liver resident cells. The PKH26^+^ proportion of each population is shown. Data are representative of two independent experiments with at least 4 mice/group. (C) Uptake of acetylated LDL as assessed by flow cytometry within F4/80^hi^ CD11b^lo^ GFP^+^ BM- or GFP^-^ YS-derived KCs expressed as a percentage of each population.

We next set out to determine if both KC populations were comparable in a range of functional assays. First we studied another essential function of KCs, namely the clearance of effete red blood cells (RBCs). We injected neuraminidase treated PHK-26-labelled RBCs [29] intravenously into chimeric mice and assessed their uptake by BM-derived or YS-derived KC populations by flow cytometry. These data showed that at 2 weeks post-irradiation, although still at low frequencies in the liver (Fig. 1), differentiated BM-derived KCs were capable of phagocytosing RBCs with the same efficiency as their YS-derived counterparts (Fig. 3A). A similar pattern was observed at 6 weeks post-irradiation (Fig. 3B). Hence, differentiation to allow for this essential KC function occurs rapidly once BM-derived cells enter the liver microenvironment.

### YS-Derived macrophages more efficiently accumulate acetylated low density lipoproteins

Given that YS-derived macrophages had a scavenger phenotype, we next utilised the BM chimera model to examine the uptake of acetylated low density lipoprotein (Ac-LDL) by BM- and YS-derived liver macrophages. Hepatic mononuclear cells from BM chimeric mice were isolated and incubated with fluorescently labelled Ac-LDL. The proportion of YS-derived macrophages that were positive for LDL accumulation was significantly higher than that for BM-derived macrophages (Fig. 3C), consistent with the differential expression of scavenger receptors in these two populations.

### *In vivo* response of KC to LPS challenge

**Fig. 4:**
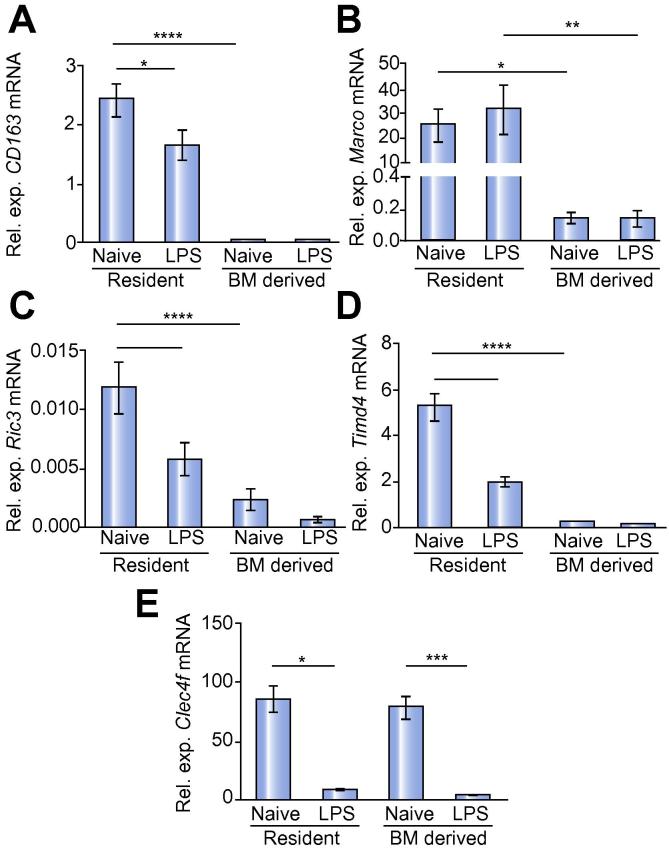
**LPS responsiveness of YS- and BM-derived KCs.** CD45.1/.2 chimeras that were 6 weeks post-irradiation were treated with 100 μg of LPS or sham treated and the KCs isolated 24 h later. Groups of 8 chimeric mice were treated and 2 livers pooled to make 4 individual replicates per treatment group. KCs were sorted into YS- and BM-derived populations based on expression of CD45.1, F4/80, and CRIg. (A) Relative expression of *CD163*, (B) *Marco*, (C) *Ric3*, (D) *Timd4* and (E) *Clec4f*. Data are pooled from 2 separate experiments. Data were tested for normal distribution and then analysed using a one-way ANOVA with post-test. ∗∗∗∗*p* <0.0001, ∗∗∗*p* <0.001, ∗∗*p* <0.01, ∗*p* <0.05.

KCs play a major role in the innate immune response to infection and are continually conditioned by endotoxin draining from the intestinal tract [30], [31], [32]. Previous *in vitro* studies have demonstrated that multiple exposure to LPS may lead to a state of LPS tolerance, wherein a second exposure to LPS fails to induce gene expression to a similar extent as the primary exposure [33]. Furthermore, Medzhitov and colleagues have argued that LPS inducible genes can be classified as “tolerizable” or non-tolerizable”, reflecting different epigenetic regulation of transcription [34]. It might be expected that KCs had mechanisms to avoid loss of function due to LPS tolerance and that this might represent one aspect of tissue specific conditioning [15]. We therefore asked whether genes that were not expressed by BM-derived KCs in the steady state were LPS inducible. We treated bone marrow chimeric mice with LPS, sorted the BM-derived and YS-derived KCs 24 h later and used real-time PCR to analyse mRNA abundance for a selected panel of genes. *Marco* is an example of a non-tolerizable gene and is expressed exclusively in YS-derived KCs (Fig. 4A). LPS treatment had no effect on *Marco* mRNA levels in either YS-derived or BM-derived KCs (Fig. 4A) suggesting that the differences observed in baseline levels of *Marco* mRNA were not a result of differences in long-term LPS exposure between the two populations. LPS treatment resulted in a down-regulation of *Cd163* mRNA abundance in YS-derived KCs, but had no effect on the minimal abundance of *Cd163* mRNA in BM-derived KCs (Fig. 4B). Similarly, LPS treatment reduced the abundance of *Ric3* and *Timd4* mRNA in YS-derived KCs and decreased *Clec4f* mRNA abundance in both populations of KCs (Fig. 4C–E). These data indicate that even high dose LPS exposure does not drive the final differentiation of BM-derived KCs to match the gene expression profile of YS-derived KCs, at least for the genes assayed here, and it appears that both populations are capable of responding to LPS challenge.

### YS-derived and BM-derived KCs show differential uptake of bacteria pathogens

**Fig. 5:**
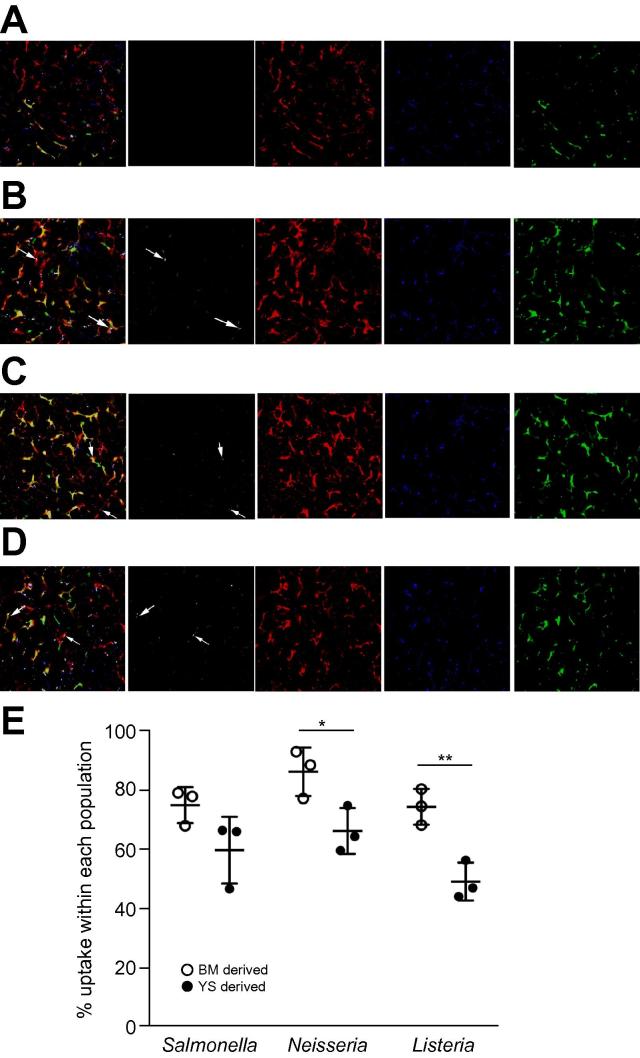
**Uptake of bacterial species by BM- and YS-derived KCs.** Two-photon imaging of live liver tissue from B6.MacGreen → CD45.1 chimeras from (A) control mice or mice that were injected with heat-killed, Syto 62 labelled (B) *S. typhimurium*, (C) *Neisseria meningitidis* or (D) *Listeria monocytogenes*. (E) Quantification of bacterial uptake by F4/80^hi^ CD11b^lo^ GFP^+^ BM- or GFP^-^ YS-derived KCs by flow cytometry.

Given that KCs are central to the clearance of systemic bacteria and MARCO has been associated with bacterial uptake [35], [36], [37], we next assessed the ability of YS- and BM-derived macrophages to take up three different bacterial pathogens. For these experiments, we generated B6.MacGreen → B6.CD45.1 BM chimeras and injected fluorescently labelled, heat-killed *Salmonella enterica* subspecies *enterica* serovar typhimurium (*S*. typhimurium), *Neisseria meningitidis* or *Listeria monocytogenes*. Phagocytosis was assessed 2 h after intravenous injection of bacteria by flow cytometry on isolated hepatic monocytes (MNCs) or by fluorescence microscopy on whole liver tissue (Fig. 5). Unexpectedly, our data showed that a greater proportion of BM-derived KCs phagocytosed *N. meningitidis* and *L. monocytogenes*, compared to YS-derived KC, with a similar trend also observed for *S. typhimurium* (Fig. 5E). Collectively with our data above on erythrophagocytosis, we conclude that whilst phagocytosis per se is not a unique property of either KC population, differences in phagocytic clearance rate based on ligand specificity exist.

### YS-derived and BM-derived KCs respond similarly to *Leishmania* infection

**Fig. 6:**
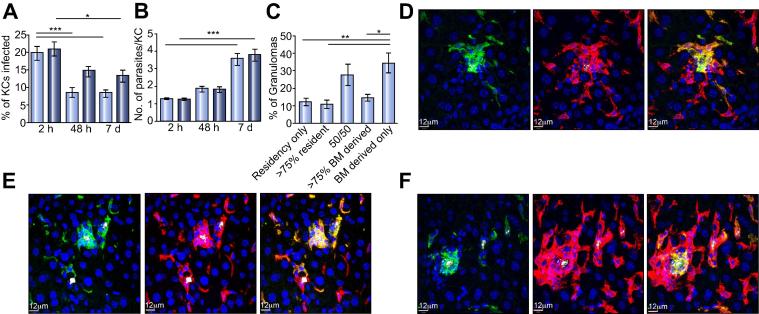
**Control of *Leishmania* infection by YS- and BM-derived KCs.** (A) Percentage of liver BM-derived (light blue bars) and liver resident (dark blue bars) KCs that are infected at 2 h, 48 h and 7 days post-infection with *L. donovani*. (B) The number of parasites per cell in BM-derived (light blue bars) and liver resident KCs (dark blue bars). (C) The percentage of inflammatory foci formed at 7 days post-infection that are made up of resident or BM-derived KCs. Data were analysed via Kruskal-Wallis test. ∗*p* <0.05, ∗∗∗*p* <0.001. (D) Immunofluorescent images demonstrating an inflammatory focus predominantly made up of liver resident KCs (green). F4/80 (red), *L. donovani* (white). (E) Immunofluorescent images demonstrating an inflammatory focus predominantly made up of BM-derived KCs (green). F4/80 (red), *L. donovani* (white). (F) Immunofluorescent images demonstrating an inflammatory focus made up of a mixture of liver resident (green) and BM-derived KCs (red). F4/80 (red), *L. donovani* (white).

Finally, we evaluated the ability of these two KC populations to respond to and contain infectious challenge by a KC-tropic intracellular parasite. *Leishmania donovani* infection of mice via the intravenous route leads to rapid KC infection and the subsequent T cell-dependent generation of inflammatory foci termed granulomas [23], [38], [39]. We infected reciprocal BM chimeric mice with transgenic tdTomato-expressing *L. donovani* amastigotes, and determined the proportion of KCs of each origin that were infected. Unlike the situation observed with bacterial uptake (Fig. 5), YS-derived and BM-derived KCs did not differ in their ability to phagocytose *L. donovani*, (Fig. 6A). Both populations were capable of killing parasites, as judged by a decrease in the percentage of infected cells observed by 48 h post-injection (Fig. 6A), though this difference only reached significance for BM-derived KCs. The difference between BM-derived and YS-derived KCs at 48 h post-infection was not significant (Fig. 6A) suggesting that both populations are capable of controlling infection with *L. donovani*. By 7 days post-infection, both KC populations showed a reduction in percentage of infected cells when compared to the 2 h time point (Fig. 6A), indicating a similar level of control of infection.

Furthermore, quantifying the number of parasites within each infected KC showed that BM-derived and YS-derived KCs phagocytosed the same number of parasites at 2 h post-injection and had equal numbers of parasites per cell by 7 days post-infection (Fig. 6B). These data, in conjunction with those shown in Fig. 6A suggest that those KCs that do not clear infection are able to similarly support parasite multiplication and an increase in the mean number of parasites present per cell (Fig. 6B).

As an additional measure of function, we examined the ability of these two populations of KCs to become the focus for granuloma formation. Inflammatory foci were scored into 5 categories: foci that contained only YS-derived KCs, mostly YS-derived KCs a 50:50 mix of YS- and BM-derived KCs, mostly BM-derived KCs and only BM- derived KCs. By this analysis, we were able to show that inflammatory foci were more likely to be formed around BM-derived KCs than around YS-derived KCs (Fig. 5C), although both KC populations were capable of seeding granuloma formation. Examples of granulomas from YS-derived KCs (Fig. 5D), BM-derived KCs (Fig. 5E) and mixed granulomas (Fig. 5F), were evident in all infected mice.

In summary, our data collectively argue that BM-derived KC are not only capable of differentiating in the liver to look and act like YS-derived KCs but are also capable of responding to an infectious challenge in a similar way.

## Discussion

The recent investigations into the origins of tissue macrophages in mice have demonstrated that in the lung, spleen, skin, pancreas, kidney and liver, tissue-resident macrophages are not derived from haematopoietic precursors in the steady state, but are seeded in embryonic tissues (YS-derived) and self-maintain locally in adult tissues [5], [8], [9]. We have now shown that following irradiation induced liver damage, YS-derived KCs are partially replaced by BM-derived precursors, and that these cells differentiate in the liver into mature KCs, where they become resident and share >99% of their gene expression with YS-derived KCs. Although functionally distinct regarding Ac-LDL uptake (YS >BM) and phagocytosis of bacteria (BM >YS), these newly differentiated, BM-derived KCs are equally capable of responding to soluble (LPS) and parasitic insult as YS-derived KCs and to equally perform essential housekeeping functions like RBC clearance. We have also identified a combination of phenotypic markers including Marco and Tim4 that along with well characterised molecules including CRIg and F4/80 can be used to phenotypically separate liver macrophages that are recently BM-derived or of YS-origin.

The recent investigations and characterisation of Clec4f through gene knockout and sequencing approaches [15], [40] have confirmed Clec4f as a KC-restricted molecule, making it a good candidate molecule for defining KC differentiation. These investigations have now been further expanded by Scott *et al.* to include specific depletion of KC in the liver through the use of KC-DTR mice in which the human DTR gene was inserted into the 3’ untranslated region of the *Clec4f* gene. Administration of diphtheria toxin (DT) resulted in specific depletion of KCs in the liver [18]. We found that BM-derived KCs up regulate *Clec4f* gene expression and take up their characteristic stationary sinusoidal position, becoming KCs by all morphologic and dynamic definitions that are currently available. In their recent study, Lavin *et al.* described steady state (and presumably mostly YS-derived) KC as most closely associated with red pulp macrophages of the spleen, in terms of gene expression in these two populations. It is noteworthy, therefore, that YS-derived KCs, but not BM-derived KCs in our study have abundant mRNA for *Ric3*, *CD163*, *St6galnac2*, *Epor*, *Hmox1* and *Ecm1*, genes also reported in the ImmGen database as highly expressed in red pulp macrophages. These data suggest that full acquisition of housekeeping properties conserved between splenic and YS-derived KCs has not yet occurred in the BM-derived KCs we have studied.

Whilst this manuscript was under review, Scott *et al.* published gene signatures distinguishing YS-derived KCs from BM-derived KCs obtained at different times post DTR-mediated depletion of YS-derived KCs [18]. Their data show many similarities, but some differences with that presented here. Of note, however, five (*Timd4*, *Colec12*, *Cd163*, *Snrpn* and *Xlr*) of the twelve genes described as differentially expressed in YS but not BM-derived KCs in their study [18] were also identified by us, providing confidence in their assignment independent of methodology used.

In addition to the 5 commonly identified genes associated with YS-derived KCs, we also identified a further 37 transcripts that were differentially expressed. Analysis of these showed enrichment in YS-derived KCs for GO terms related to ion homeostasis, and differential expression of genes associated with red blood cell phagocytosis and turnover. However, our data using an *in vivo* phagocytosis assay demonstrated no difference in the ability of BM-derived and YS-derived KCs to phagocytose labelled red blood cells, suggesting that the two populations were equally capable of contributing to this important KC function [41]. Nevertheless, further studies will be required to determine whether differences exist between the two populations in the scavenging of haemoglobin and the break down and recycling of red blood cells.

It was of significant note that there were no genes expressed by BM-derived cells that were not expressed by YS-derived KCs. This is in contrast to the findings of Scott *et al.* who found two genes (*Ccr3 and Tspan32*) that were uniquely expressed by bone marrow-derived KCs [18]. Together, the similarity in gene expression profiles in these two studies suggest that BM-derived monocytes are malleable in nature and responsive to the microenvironmental cues they receive, enabling them to differentiate and perform functions required by the tissue that they become resident in. It would follow that this is not hindered by extensive residual expression of a BM-specific gene signature that cannot be reprogrammed when required.

These studies confirm and further extend the observations of others, that irradiation induces a loss of a proportion of YS-derived macrophages in a number of tissues [7], [8], [42], triggering repopulation from BM-derived precursors. In non-radiation-induced depletion models, loss of a proportion of the tissue-resident macrophage population, induces local proliferation and repopulation by self-renewal in the lungs, liver, epidermis and brain [5]. In contrast, the depletion of tissue-derived macrophages via irradiation appears to trigger a different mechanism of repopulation, with long-term seeding of hematopoietic stem cell-derived cells being a predominant feature of repopulation in the spleen, lungs, peritoneum and bone marrow [8]. This difference is likely associated with the absence (conditional gene targeting) *vs.* presence (irradiation) of collateral damage and the induction of stress induced responses in a multitude of different cell types in any given organ. However, in the liver, complete loss of KCs via clodronate liposome mediated depletion also results in repopulation of KCs from BM precursors [7], implying that there may be a threshold that triggers differentiation of BM-derived cells when the niche cannot be sufficiently filled by the proliferation of the YS-derived population. This phenomenon may be a secondary safety net for the function of the liver, as the BM-derived KCs appear to be capable of differentiating *in situ* to have a very similar gene expression profile to the cells they replace, with a similar ability to respond to an infectious challenge.

The use of the BM chimera model allowed us to evaluate the functional capacity of YS- and BM-derived KCs in a uniform microenvironment, obviating any differences in global liver function that might otherwise have arisen if we had used a total replacement strategy [18]. Uptake of *E. coli in vitro* by isolated YS- and BM-derived KCs has been reported to be similar [18]. In contrast, to study KC function we used an extended battery of *in vivo* assays that retain natural microenvironmental context and that employed both soluble and particulate ligands. In addition, we used a well- characterised parasitic model that allowed us to monitor innate microbicidal activity as well as T cell-dependent host protective immunity (expressed as granulomatous inflammation). Whereas uptake of Ac-LDL was more efficient in YS-derived KCs, we found that BM-derived KCs were more phagocytic towards *L. monocytogenes*, *N. meningitides* and *S. typhimurium.* As MARCO has been implicated in uptake of each of these bacteria, this result was unexpected. The differences were not absolute, but may suggest that with increasing ligand complexity, there is greater scope for receptor redundancy and/or cooperativity, a level of complexity that is not fully captured by gene expression analysis. For example, both *N. meningitides* and *L. monocytogenes* utilise SR-A in addition to MARCO to interact with macrophages [35], a molecule that showed no difference in expression between the two macrophage populations at an RNA level in our gene expression studies.

Our data are not without clinical relevance, where radiation-induced liver damage (radiation hepatitis) can result in hepatic fibrosis. BM transplantation for treatment of haematological malignancy uses total body irradiation as a pre-conditioning strategy prior to transplantation. In this system, the remaining host macrophages [43] including KCs in the liver [44] have been implicated in the suppression of the pathological graft *vs.* host disease (GVHD) response. Whether a similar level of radiation resistance is observed in human KCs requires further investigation. Given that inflammatory events and the resultant cellular turnover are likely common in the liver *in vivo*, it is essential that BM-derived KCs have host protective functions as well as functional competence to maintain the homeostatic functions of the liver. In spite of some selected functional differences, our data provide strong evidence that this is indeed the case.

## Financial support

This work was funded by the UK Medical Research Council (Grant #G0802620) and the Australian National Health and Medical Research Council (Grant #APP1105817).

## Conflict of interest

The authors who have taken part in this study declared that they do not have anything to disclose regarding funding or conflict of interest with respect to this manuscript.

## Authors’ contribution

LB conceived and designed the study, performed experiments, analysed data and wrote the manuscript. AS, JM, TCMF, BT, FLR, KW-B, JWJM, EKL and JED performed and analysed some of the experiments. SM analysed the gene expression array data. Cre and KPM contributed to data analysis. PMK conceived and designed the study, analysed data, and wrote the manuscript.

## Figures and Tables

**Table 1:** **The genes differentially expressed between liver resident and BM derived KC.**
