# Supplementary material for: Bone marrow-derived and resident liver macrophages display unique transcriptomic signatures but similar biological functions
Source: J Hepatol. 2016 Oct;65(4):758–68. doi: 10.1016/j.jhep.2016.05.037 (PMC5028381; doi:10.1016/j.jhep.2016.05.037)
Supplement: Supplementary data 1 [file mmc1.pdf]

Bone marrow-derived and resident liver macrophages display unique transcriptomic signatures but similar biological functions

Lynette Beattie, Amy Sawtell, Jason Mann, Teija CM Frame, Bianca Teal, Fabian de Labastida Rivera, Najmeeyah Brown, Katherine Walwyn-Brown, John W.J. Moore, Sandy MacDonald, Eng-Kiat Lim, Jane E Dalton, Christian R Engwerda, Kelli P MacDonald, Paul M. Kaye

Table of contents

Supplementary materials and methods……………………………………….. 2

Supplementary Table 1…………………………………………………………. 8

References……………………………………………………………………… 13

Materials and methods:

Mice and Infection

C57BL6 or B6.CD45.1 mice were obtained from Charles River (UK) or the Australian Resource Centre (WA). mT/mG [1], lysMcre [2] and B6.MacGreen [3] mice have been previously described. Mice were bred and housed under specific pathogen-free conditions and used at 6–12 weeks of age. The Ethiopian strain of *Leishmania donovani* (LV9) and tandem Tomato fluorescent protein expressing LV9 (tdTom.LV9) [4] were maintained by serial passage in Rag-1$^{-/-}$ mice. Amastigotes were isolated from infected spleens [5], and mice were infected with 3x10^{7} *L. donovani* amastigotes intravenously (i.v.) via the tail vein in 200 µl of RPMI 1640 (GIBCO, UK). For the generation of chimeras, mice were placed on acidified water for at least 2 days prior to irradiation. Donor mice were irradiated with 1100 rads on a split-dose regimen (550 rads per dose, 24hours apart) and were then reconstituted with 2-5x10^{6} donor bone marrow cells via tail vein injection. Reconstituted mice were treated with oral antibiotics (Baytril) for 4 weeks post-reconstitution.

Liver enzyme analysis

Heparinized blood was immediately centrifuged for 10 min at 300 × *g*. Plasma was stored at -80°C until analysis. Alanine aminotransferase (ALT) and aspartate transaminase (AST) levels were determined using a Beckman Unicell DxC800 analyzer in a single batch.

Image Analysis

For 4D analysis, 20-3 5µm Z stacks were acquired with a Z distance of 2 µm approximately every 15-30sec. Data were rendered and analysed using Volocity software (Perkin Elmer) and cell analysis performed automatically with manual checking as previously described [6].

Confocal Microscopy

Confocal microscopy was performed on 20 µm frozen sections. For tissue containing tdTom expressing parasites, tissues were fixed in 4% paraformaldehyde (PFA) for two hours before overnight incubation in 30% sucrose and embedding in OCT medium (Sakura). Antibodies were conjugated to Alexa488 or Alexa647 (eBioscience, UK). Slides were blinded before imaging on a Zeiss LSM510 axioplan microscope (Carl Zeiss Microimaging). Data were rendered and analysed using Volocity software (Improvision).

Flow cytometry and cell sorting

Hepatic mononuclear cells were prepared from the livers of chimeric mice. 20 chimeric mice were used for the microarray study. We conducted five independent sorts to generate samples for analysis. For each sorting experiment (n=4 mice), we isolated both YS-derived and BM-derived KC from liver mononuclear cells that were pooled prior to sorting. 3 of the experiments were performed on chimeric mice that contained GFP^+ liver resident KCs and 2 experiments were performed on chimeric mice that contained GFP^+ BM derived KCs. To isolate liver mononuclear cells, livers were perfused with 0.4mg/mL of warmed liberase TL (Roche) then removed,

homogenised with a scalpel blade and digested at 37°C for 45min. Digested livers were passed over 100 µm cell strainers, washed in PBS containing 2%FCS and resuspended in 33% percoll for centrifugation at 693g for 12min. The remaining cell pellet was kept for further analysis. Isolated cells were blocked with anti CD16/32 then labelled with F4/80-Alexa488, GR-1 PeCy7 (eBioscience) and CRIg (clone 14G6, Helmy Cell 2006, provided by Genentech Inc) that was conjugated to Atto 647H via lightning-link conjugation (Innova Biosciences). Cells were sorted on a MoFlo cell sorter (DAKO) based on expression of cell surface markers and GFP. Sorted cells were pelleted and resuspended in Trizol (Invitrogen). A small fraction of cells (approximately 3000 sorted cells) were spun onto glass slides, fixed in methanol and stained with Giemsa for morphological analysis.

Real-time PCR

Purified cells were resuspended and frozen in QIAZOL lysis buffer. RNA was isolated using a miRNA easy kit (QIAGEN). RNA was reverse transcribed into cDNA with a Superscript III first-strand synthesis kit (Invitrogen). Real-time quantitative PCR was performed with Fast SYBR green PCR master mix in a StepOne Plus Real-Time PCR system (Applied Biosystems). Accumulation of target genes was normalized to HPRT, (results were the same if normalised to β-actin [7] or ribosomal protein L32 [8]) and expressed as relative expression via the change in cycle threshold (ΔΔCT) analysis method (relative expression in test sample versus RPMI controls). Oligonucleotides used for the specific amplification of target genes were; Timd-4: AGAATGTGCGCTTGGAGCTGAG/GGTTGGGAGAACAGATGTGGTC, CD163: GGCTAGACGAAGTCATCTGCAC/CTTCGTTGGTCAGCCTCAGAGA,

Colec12: ACTCCAAGCACGGTCAGCTCAT/CTTGTTGCCAGTTGGACCAGGT, Ric3: GGTAGTGGAAGAGGACTGATGG/AGTGGAGCAGTTCCGATCCTCT, Clec4f: TCACAGCCTTGGAGACCTGAGT/CCTAAGCCTCTGGATAGCCACT, MARCO: ATGGCACCAAGGGAGACAAAGG/GCCTGGTTTTCCAGCATCACCT.

RBC Phagocytosis

BM chimeric mice were generated as described above. Two or six weeks post irradiation and bone marrow transplantation, desialyated and labelled red blood cells were transferred IV as described [9]. Briefly, normal mouse red blood cells were labelled with 2-mM solution of PKH-26 (Sigma) for 5min at 25°C. The labelled cells were then treated with 0.012U of *Arthrobacter ureafaciens* and *Vibrio cholera* neuraminidases (Sigma) for 1hr at 37°C. A chimeric mouse that received no red blood cell injection was used as a control.

LPS Treatment

BM chimeric mice were generated as described above with C57BL/6 recipients and B6.CD45.1 donors. Six weeks after bone marrow transplant, the recipient mice were injected with 100ug LPS (Sigma) intravenously. 24hrs post-injection, the mice were sacrificed, and the livers prepared for cell sorting as described above. KCs were sorted based on expression of CD45.1/.2, CRIg and F4/80.

In Vivo Bacterial Uptake Assay

Salmonella enterica subspecies *enterica* serovar Typhimurium (*S*. typhimurium), *Neisseria meningitidis* or *Listeria monocytogenes* were grown to an optical density of 0.8 and 600nm and then washed and heat-killed for 2hrs at 60°C and stored at 4°C until use. Heat-killed bacteria were labelled with SYTO 62 (Molecular Probes) for 30 mins at RT. Labelled bacteria were injected intravenously into the tail vein of B6.MacGreen → B6.CD45.1 chimeric mice, and the livers removed after 2 hours. Whole, unfixed liver tissue was imaged on a Zeiss 780 NLO 2-photon microscope (Carl Zeiss Microimaging). Images were acquired with a 40x 1.1 water immersion objective and fluorescence excitation provided by a Chameleon XR Ti:sapphire laser (Coherent) tuned to 900nm. For quantitation via flow cytometry, hepatic mononuclear cells were prepared and samples labelled with F4/80 (PE) (BioLegend) before acquisition using a BD LSR Fortessa (BD). SYTO 62 was detected in the APC (R670/14) channel. Data was analysed using FlowJo v10 OSX software (Treestar, Oregon).

Uptake of acetylated LDL

Hepatic mononuclear cells were prepared from B6.MacGreen → B6.CD45.1 chimeric mice (8 mice, 4 pools of 2 livers) and depleted of B and T cells using EasySep mouse biotin selection kit (Stemcell Technologies). Samples were labelled with F4/80 (PE-Cy7), CD11b (PerCP-Cy5.5), CD45.1 (PE), CD45.2 (AF700) and Tim4 (AF647) and then incubated with 10µg/mL Alexa Flour 594 AcLDL (Molecular Probes) for 1 hr at 37°C. Flow cytometry acquisition was performed using a BD LSR

Fortessa (BD) and data was analysed using FlowJo v10 OSX software (Treestar, Oregon).

Supplementary Table 1:

Gene Ontology terms over-represented in the analysis of genes expressed by YS-derived but not BM-derived KCs.

GO ACCESSION	GO Term	p-value	Count in Selection
GO:0055065	metal ion homeostasis	0.0002	5
GO:0055080	cation homeostasis	0.0003	5
GO:0034394	protein localization to cell surface	0.0003	2
GO:0050801	ion homeostasis	0.0004	5
GO:0006875	cellular metal ion homeostasis	0.0010	4
GO:0006897|GO:0016193|GO:0016196	endocytosis	0.0014	4
GO:0030003	cellular cation homeostasis	0.0014	4
GO:0004900	erythropoietin receptor activity	0.0015	1
GO:0004411	homogentisate 1,2-dioxygenase activity	0.0015	1
GO:0033292	T-tubule organization	0.0015	1
GO:0006873	cellular ion homeostasis	0.0017	4
GO:0005044	scavenger receptor activity	0.0018	2
GO:0048878	chemical homeostasis	0.0019	5
GO:0055082	cellular chemical homeostasis	0.0023	4
GO:0006788	heme oxidation	0.0030	1
GO:0031783	type 5 melanocortin receptor	0.0030	1

	binding		
GO:0086036	regulation of cardiac muscle cell membrane potential	0.0030	1
GO:0005686	U2 snRNP	0.0030	1
GO:0036017	response to erythropoietin	0.0030	1
GO:0004392	heme oxygenase (decyclizing) activity	0.0030	1
GO:0036018	cellular response to erythropoietin	0.0030	1
GO:0032764	negative regulation of mast cell cytokine production	0.0030	1
GO:0038162	erythropoietin-mediated signaling pathway	0.0030	1
GO:0070436	Grb2-EGFR complex	0.0030	1
GO:0031780|GO:0071856	corticotropin hormone receptor binding	0.0030	1
GO:0055072	iron ion homeostasis	0.0032	2
GO:0038024	cargo receptor activity	0.0035	2
GO:0015891|GO:0015892	siderophore transport	0.0045	1
GO:0032763	regulation of mast cell cytokine production	0.0045	1
GO:0015688	iron chelate transport	0.0045	1
GO:0070996	type 1 melanocortin receptor binding	0.0045	1

GO:0033212	iron assimilation	0.0045	1
GO:0042167	heme catabolic process	0.0045	1
GO:0033214	iron assimilation by chelation and transport	0.0045	1
GO:0004566	beta-glucuronidase activity	0.0045	1
GO:0046149	pigment catabolic process	0.0045	1
GO:0019725	cellular homeostasis	0.0047	4
GO:0006909	phagocytosis	0.0051	2
GO:0006874	cellular calcium ion homeostasis	0.0054	3
GO:0086018	SA node cell to atrial cardiac muscle cell signalling	0.0060	1
GO:0086015	SA node cell action potential	0.0060	1
GO:0031781	type 3 melanocortin receptor binding	0.0060	1
GO:0043305	negative regulation of mast cell degranulation	0.0060	1
GO:0033015	tetrapyrrole catabolic process	0.0060	1
GO:0006787	porphyrin-containing compound catabolic process	0.0060	1
GO:0031782	type 4 melanocortin receptor binding	0.0060	1
GO:0031779	melanocortin receptor binding	0.0060	1
GO:0055074	calcium ion homeostasis	0.0062	3
GO:0072503	cellular divalent inorganic cation	0.0063	3

	homeostasis		
GO:0055076	transition metal ion homeostasis	0.0066	2
GO:0055001	muscle cell development	0.0073	2
GO:0072507	divalent inorganic cation homeostasis	0.0073	3
GO:0005134	interleukin-2 receptor binding	0.0075	1
GO:0001537	N-acetylgalactosamine 4-O-sulfotransferase activity	0.0075	1
GO:2000369	regulation of clathrin-mediated endocytosis	0.0075	1
GO:0033007	negative regulation of mast cell activation involved in immune response	0.0075	1
GO:0044183	protein binding involved in protein folding	0.0075	1
GO:0002246	wound healing involved in inflammatory response	0.0075	1
GO:0006572	tyrosine catabolic process	0.0075	1
GO:0071276	cellular response to cadmium ion	0.0075	1
GO:0005685	U1 snRNP	0.0075	1
GO:0086070	SA node cell to atrial cardiac muscle cell communication	0.0075	1
GO:0045766	positive regulation of angiogenesis	0.0080	2
GO:0005581	collagen	0.0080	2

GO:0043619	regulation of transcription from RNA polymerase II promoter in response to oxidative stress	0.0090	1
GO:0006559	L-phenylalanine catabolic process	0.0090	1
GO:0071243	cellular response to arsenic-containing substance	0.0090	1
GO:1902222	erythrose 4-phosphate/phosphoenolpyruvate family amino acid catabolic process	0.0090	1
GO:0004630	phospholipase D activity	0.0090	1
GO:0006954	inflammatory response	0.0093	3

References

[1] Muzumdar MD, Tasic B, Miyamichi K, Li L, Luo L. A global double-fluorescent Cre reporter mouse. Genesis 2007;45:593-605.

[2] Clausen BE, Burkhardt C, Reith W, Renkawitz R, Forster I. Conditional gene targeting in macrophages and granulocytes using LysMcre mice. Transgenic Res 1999;8:265-277.

[3] Sasmono RT, Oceandy D, Pollard JW, Tong W, Pavli P, Wainwright BJ, et al. A macrophage colony-stimulating factor receptor-green fluorescent protein transgene is expressed throughout the mononuclear phagocyte system of the mouse. Blood 2003;101:1155-1163.

[4] Beattie L, Peltan A, Maroof A, Kirby A, Brown N, Coles M, et al. Dynamic imaging of experimental Leishmania donovani-induced hepatic granulomas detects Kupffer cell-restricted antigen presentation to antigen-specific CD8 T cells. PLoS pathogens 2010;6:e1000805.

[5] Smelt SC, Engwerda CR, McCrossen M, Kaye PM. Destruction of follicular dendritic cells during chronic visceral leishmaniasis. J Immunol 1997;158:3813-3821.

[6] Beattie L, d'El-Rei Hermida M, Moore JW, Maroof A, Brown N, Lagos D, et al. A transcriptomic network identified in uninfected macrophages responding to inflammation controls intracellular pathogen survival. Cell host & microbe 2013;14:357-368.

[7] Shih VF, Davis-Turak J, Macal M, Huang JQ, Ponomarenko J, Kearns JD, et al. Control of RelB during dendritic cell activation integrates canonical and noncanonical NF-kappaB pathways. Nature immunology 2012;13:1162-1170.

[8] Reis BS, Rogoz A, Costa-Pinto FA, Taniuchi I, Mucida D. Mutual expression of the transcription factors Runx3 and ThPOK regulates intestinal CD4(+) T cell immunity. Nature immunology 2013;14:271-280.
[9] Bratosin D, Estaquier J, Ameisen JC, Aminoff D, Montreuil J. Flow cytometric approach to the study of erythrophagocytosis: evidence for an alternative immunoglobulin-independent pathway in agammaglobulinemic mice. Journal of immunological methods 2002;265:133-143.
