# Supplementary figures and images for: Bone marrow-derived and resident liver macrophages display unique transcriptomic signatures but similar biological functions
Source: J Hepatol. 2016 Oct;65(4):758–68. doi: 10.1016/j.jhep.2016.05.037 (PMC5028381; doi:10.1016/j.jhep.2016.05.037)

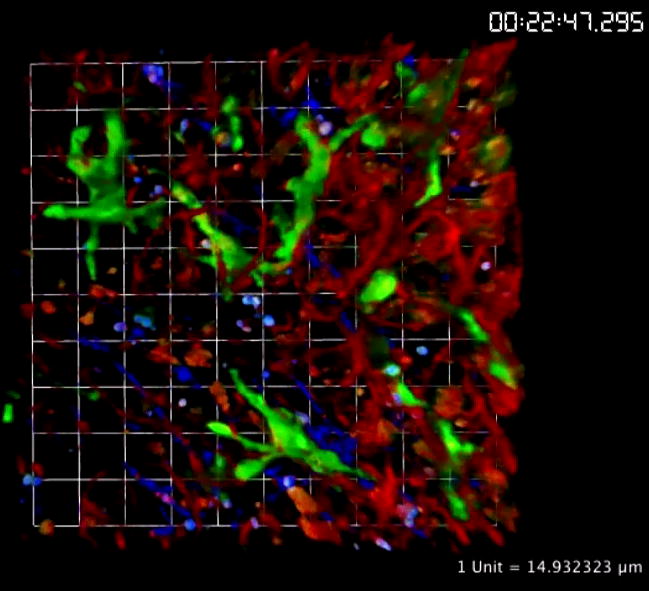

Supplement: Supplementary movie 1 [file mmc3.jpg]

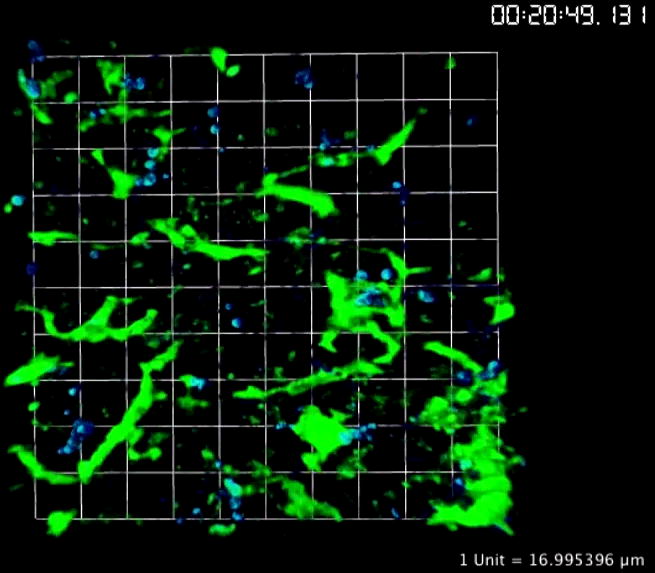

Supplement: Supplementary movie 2 [file mmc4.jpg]
